# Damage Identification of Large Generator Stator Insulation Based on PZT Sensor Systems and Hybrid Features of Lamb Waves

**DOI:** 10.3390/s18092745

**Published:** 2018-08-21

**Authors:** Ruihua Li, Hao Li, Bo Hu

**Affiliations:** 1 Department of Electrical Engineering, Tongji University, Shanghai 201804, China; bobo_hu@tongji.edu.cn; 2 College of Electrical Engineering, Shanghai University of Electrical Power, Shanghai 200090, China; hlipower@shiep.edu.cn

**Keywords:** rotating machine insulation, rotating machine insulation testing, crack detection, acoustic signal processing, acoustic applications

## Abstract

Large generators are the principal pieces of equipment in power systems, and their operation reliability critically depends on the stator insulation. Damages in stator insulation will gradually lead to the failure and breakdown of generator. Due to the advantages of Lamb waves in Structural health monitoring (SHM), in this study, a distributed piezoelectric (PZT) sensor system and hybrid features of the Lamb waves are introduced to identify stator insulation damage of large generator. A hierarchical probability damage-imaging (PDI) algorithm is proposed to tackle the material inhomogeneity and anisotropy of the stator insulation. The proposed method includes three steps: global detection using correlation coefficients, local detection using Time of flight (ToF) along with the amplitude of damage-scattered Lamb wave, and final images fusion. Wavelet Transform was used to extract the ToF of Lamb wave in terms of the time-frequency domain. Finite Element Modeling (FEM) simulation and experimental work were carried out to identify four typical stator insulation damages for validation, including inner void, inner delamination, puncture, and crack. Results show that the proposed method can precisely identify the location of stator insulation damage, and the reconstruction image can be used to identify the size of stator insulation damage.

## 1. Introduction

Stator winding insulation is the component most related to the reliability of a high voltage (HV) rotating machines [1,2,3]. In long-term service, stator insulation is exposed to a combination of thermal, electrical, and thermal-mechanical stresses. These multiple stresses cause insulation aging. Previous research has shown that the invalidation of stator insulation in the aging progress is the root cause for large generator breakdown [4,5]. Implementing condition monitoring (CM) technology on stator insulation holds the promise of enhancing the safety and economic operation of a large generator.

There has been considerable effort over recent decades to identify the causes of stator insulation degradation and find methods for assessing the condition of stator insulation [6]. Various non-destructive methods have been proposed for CM of stator insulation. Nearly all of the previous works relied on electrical measurements [5], and a plurality of researchers centered on partial discharge (PD) tests [6,7]. The aging condition of stator insulation can be indicated by means of the presented aging-related electrical characteristics. These electrical indicators are well applied for CM and fault diagnosis of stator insulation; however, currently, the relationship between these electrical characteristics and the micro insulation damage is still not clear enough. In fact, the inception and development of insulation damages, such as void, crack, delamination, etc. are the primary factor in the deterioration of stator insulation of large generators [8,9,10,11,12]. With existing CM methods, it is difficult to detect the specific features of stator insulation damage, e.g., location, type, size, etc. From an industrial application point of view, it is of great significance to detect stator insulation damage with the identification of location and size characteristics. This valuable information would help us to better understand the aging mechanisms of stator insulation. Meanwhile, it can provide an effective and reliable reference for the diagnosis of insulation status and life assessment of large generators.

Guided waves show good sensitivity to structural damage during propagation [13]. The Lamb wave, as a modality of the guided wave, has prominent advantages in both resolution, practicality, and the ability to detect the composite structure [14]. Structure health monitoring (SHM) based on the Lamb wave have been widely used to enhance the reliability of safety-critical and mission-critical equipment. Recently, there has been an increasing interest in developing imaging techniques with Lamb wave-based SHM [15,16,17]. The main concept behind this method is to detect damage graphically and intuitively by integrating Lamb wave features that are damage-sensitive. Zhao [17] provided a comprehensive review on state of art Lamb wave tomography methods for SHM, describing both the advantages, drawbacks, and practicability. Out of them, probability damage imaging (PDI) is flexible, with sparse transducers performing high quality and rapid image reconstruction. The Lamb wave-based PDI methods can not only indicate the presence of damage, but can also locate [18] and identify the severity of damage [19].

Due to the merits of Lamb wave on SHM of composite, the Lamb wave tomography method shows a potential solution for intuitively detecting stator insulation damages [20]. However, the stator insulation of large generators is mostly composed of mica tapes and epoxy resins, which can be regarded as composite materials with bar-like structures [4]. Different from other homogenous and isotropic structures, Lamb waves propagate more complexly on stator insulation. Many forms of interference affect the target feature extraction for damage imaging. In summary, there are three main challenges.

- Interference by wave dispersive and multi-modes. The inhomogeneous and anisotropy property of mica-epoxy insulation material aggravate the dispersive and multi-modes of Lamb wave [13,16].
- Interference by multi-interfaces wave reflection and attenuation. The boundaries of bar-like structure and multi-interfaces of laminate stator insulation bring unwanted wave reflections and attenuation for Lamb wave propagation.
- Environmental Interference. Noise interference from the on-site environment may reduce signal to noise ratio (SNR) of the monitored Lamb wave signal.

As mentioned, these multiple interferences represent huge obstacles to effective and accurate feature extraction in PDI procedures; as a result, they restrict the effectiveness of damage detection performance of the Lamb wave-based PDI method. To address these problems, this paper proposes an enhanced hierarchical PDI method by integrating multi-features of Lamb waves. The effects of the insulation structure complexity, as well as the anisotropy of insulation material, is alleviated by multi-feature fusion, and the anti-interference performance of the method is discussed. Finally, identification of four typical insulation damages, i.e., void, delamination, puncture, and crack are carried out by FEM simulations and experiments for validation.

## 2. Damage Identification Method of Stator Insulation Using Lamb Waves

### 2.1. Principle of Hierarchical PDI Method

**Figure 1:**
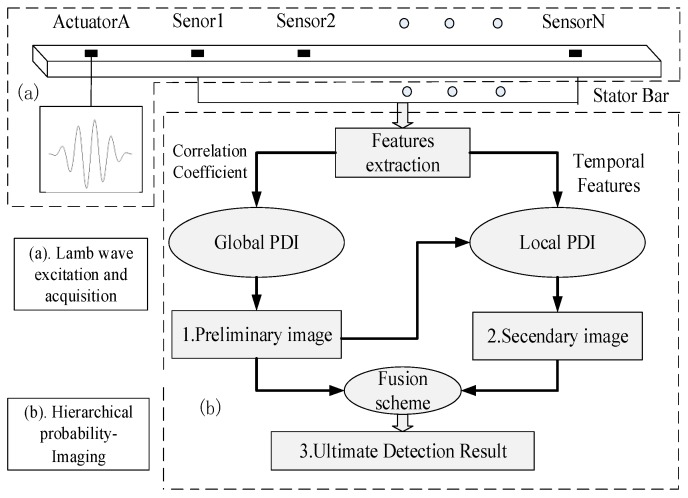
Schematic of the proposed PDI method based on hybrid features of the Lamb waves.

To visualize the damage condition (both the damage location and size) of stator insulation in an intuitive manner, the PDI method is used to represent a damage event in a 2-D binary color-scale image. Each pixel corresponds exclusively to spatial point in the inspected structure area, and the field value is linked to the probability of damage presence at that spatial position [16]. The principle of the proposed method for identification of stator insulation damage is shown in Figure 1.

The portion marked (a) in Figure 1 is the sensor network configured on the surface of stator bar. The sensor system consists of PZT wafers, where each PZT can act as both actuator and sensor for excitation and measurement of Lamb wave signals, respectively. Hybrid features of Lamb waves are extracted and integrated into a hierarchical PDI procedure. As shown in portion (b) of Figure 1, the correlation coefficient between the health and damage state of the Lamb wave signal is first extracted in global PDI to preliminarily determine damage level of stator insulation. Next, Time of Flight (ToF) and peak amplitude of damage scattered Lamb wave are extracted for local PDI to further locate the stator insulation damage. Finally, image fusion of the two above results is carried out for enhanced reconstruction of stator insulation damage condition; the detailed hierarchical PDI method is described in the following section.

### 2.2. Global PDI Method Using Correlation Coefficient as Damage Feature

The global PDI method uses a correlation coefficient as a damage feature to preliminarily image the damage condition of stator insulation. When damage exists in the propagation path, the Lamb wave signal will change: the higher the damage degree, the larger the signal variation. Correlation coefficients can quantitatively describe the variation of Lamb wave signals between health and damage states [21], which is widely used as a damage-sensitive feature to evaluate the structure’s condition. Considering one actuator/sensor path, Lamb wave signals on health state is $H=\{H_{1},H_{2},\cdots ,H_{n}\}$, and Lamb wave signals on the damaged state are $D=\{D_{1},D_{2},\cdots ,D_{n}\}$, respectively. Then, correlation coefficient of H and D is calculated as [21]:

(1) $$\rho_{H,D}=\frac{\sum_{i=1}^{n}\left(H_{i}-\mu_{H})(D_{i}-\mu_{D}\right)}{\sqrt{\sum_{i=1}^{n}{\left(H_{i}-\mu_{H}\right)}_{}^{2}}*\sqrt{\sum_{i=1}^{n}{\left(D_{i}-\mu_{D}\right)}_{}^{2}}}$$

where $\mu_{H}$ and $\mu_{D}$ are the average value of H and D respectively. The higher the damage degree, the lower correlation between signals H and D, leading to a smaller values of $\rho_{H,D}$. Then, a model of the distribution of DPP P(x,y) in stator insulation can be established [21]:

(2) $$P(x,y)=(1-\rho_{k})\cdot [\frac{\beta -R(x,y)}{\beta -1}]$$

(3) $$R(x,y)=\left\{\begin{array}{ll} R_{k}(x,y), & R_{k}(x,y)<\beta \\ \beta , & R_{k}(x,y)\geq \beta \end{array}\right\}$$

(4) $$R_{k}(x,y)=\frac{D_{ak}+D_{sk}}{D_{k}}$$

where $\rho_{k}$ is the calculated correlation coefficient; β is a scaling parameter controlling the size of damage sensitive zone; $R_{k}(x,y)$ is the relative distance between the position (x,y) to the Lamb wave sensing path, $D_{k}$ is the distance between actuator and sensor, $D_{ak}$ and $D_{sk}$ are the distance between position (x,y) to the actuator and the sensor, respectively. A damage reconstruction image can then be obtained by Equation (2).

### 2.3. Local PDI Method Using Temporal Features of Scattered Waves

**Figure 2:**
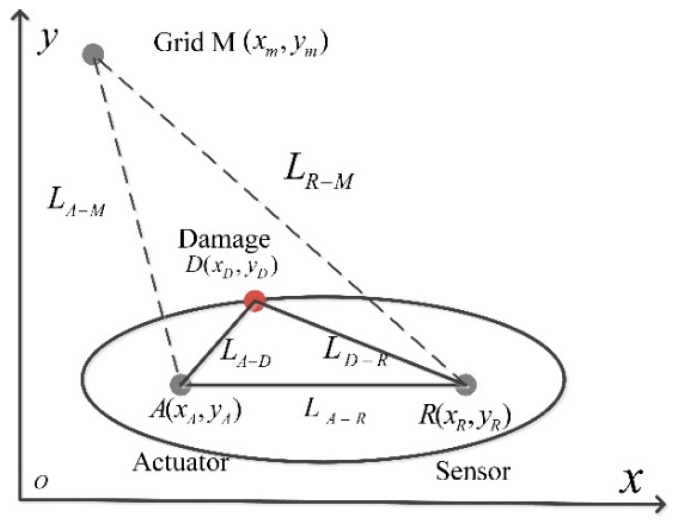
Schematic of damage localization base on ToF.

In order to further obtain the insulation damage location, local PDI based on the temporal features of scattered Lamb wave is used to enhance the image quality. According to the Huygens’ Principle, the Lamb wave scatters if it encounters damage during propagation, forming the so-called scattered Lamb wave [22]. Time of flight (ToF) of scattered Lamb wave is an effective feature to represent damage information. The principle of damage localization based on ToF is shown in Figure 2.

Considering one pair of actuator/sensor path, as shown in Figure 2, the Lamb wave is excited by actuator $A(x_{A},y_{A})$ and received by sensor $R(x_{R},y_{R})$. The ToF of scattered Lamb wave after interacting with damage $D(x_{D},y_{D})$ is

(5) $$T_{A-D-R}=(L_{A-D}+L_{D-R})/V_{g}$$

where $L_{A-D}$ is the distance from *A* to the *D*, $L_{D-R}$ is the distance from *D* to *R*, and $V_{g}$ is the group velocity of Lamb wave. For a determined $T_{A-D-R}$, an ellipse with the actuator *A* and the sensor *R* as the two foci and major axis of $L_{A-D}{}_{}+L_{D-R}=T_{A-D-R}*V_{g}$ can be formed (see Figure 2). The ellipse indicates the possible loci of damage. Obviously, *A* node closed to the ellipse trajectory possesses higher DPP value. For an optional node *M* $\left(x_{m},y_{m}\right)$, the ToF of Lamb wave spread from actuator *A* to *M* and from *M* to sensor *R* can be calculated as $T_{M}=(L_{A-M}+L_{R-M})/V_{g}$. When M is right located at the ellipse, then $T_{M}=T_{A-D-R}$. With a larger deviation between $T_{A-D-R}$ and $T_{M}$($T_{ij}=|T_{A-D-R}-T_{M}|$), the relative distance that the node away from actual damage location is larger. $T_{ij}$ can thus model the distribution of DPP in the stator insulation by the Gaussian distribution [22].

(6) $$F(T_{ij})=\int_{-\infty }^{T_{ij}}f(T_{ij})dT_{ij}$$

where, $f(T_{i}{}_{j})=\frac{1}{\sigma \sqrt{2\pi }}\exp(-\frac{T_{i}{{}_{j}}^{2}}{2\sigma^{2}})$ is the probability density function, and σ is the standard deviation, defined as:

(7) $$\sigma =w*\frac{A_{scatter}}{A_{health}}$$

where w is weight coefficient, $A_{scatter}$ and $A_{health}$ are the envelope peak of damage-scattered Lamb wave and Lamb wave received in health state, respectively. The envelope peak can used to reflect insulation damage degree. If damage degree becomes more serious, the envelope peak of scatter Lamb wave is larger [23]. For a specific $T_{ij}$ and σ, the DPP [22] of that position is defined as:

(8) $$P(T_{ij})=1-\left|F(T_{ij})-F(-T_{ij})\right|$$

Using Equation (8), the DPP values at all spatial locations can be calculated, and then an image reflecting the damage condition in stator insulation is reconstructed.

### 2.4. ToF Extraction Based on Wavelet Transform

As mentioned, the ToF of a damage scattered wave component is key to triangulating the insulation damage in a stator bar. However, the inherent property of multiple modes and the dispersion of Lamb waves make extracting ToF a huge challenge [24]. Additionally, the multi-interfaces of the laminated composite structure of stator insulation may bring massive reflection and wave attenuation in interpreting the received Lamb wave signals.

The wavelet transform (WT) is a time-frequency domain analysis method. With WT analysis, a dynamic Lamb wave signal can be interrogated using a localized fragment to display hidden characteristics fully, such as trends, breakdown points or discontinuities, and self-similarity. Hence, WT is particularly effective to extract the objective damage-sensitive features from Lamb wave signals. The WT of a signal f(t) is expressed as:

(9) $$W(a,b)=\frac{1}{\sqrt{a}}\int_{-\infty }^{+\infty }f(t)*\Psi^{*}\left(\frac{t-b}{a}\right)dt$$

where a and b are the two constants determining the scale and time axes, respectively. $\Psi^{*}(t)$ is the complex conjugate of the orthogonal wavelet function Ψ(t). Daubechies wavelet (db10) is selected as wavelet function in this paper. W(a,b) is termed the WT coefficient. It depicts the energy distribution of f(t) over the time-scale domain, and the energy spectrum is given as:

(10) $$E={\int_{b\geq 0}^{+\infty }\int_{a\geq 0}^{+\infty }\left|W(a,b)\right|}^{2}\cdot da\cdot db$$

The Lamb wave propagation can be regarded as the transportation of the energy contained in the wave packet. Thus, the ToF should be ascertained in terms of the time difference between the moments at which the wave packet reaches its maximum in the energy spectrum over the time-frequency domain. The time corresponding to the peak of the energy packet determined in terms of Equation (10) can be used to determine the actual ToF of the wave packet.

### 2.5. Image Fusion Scheme

In the above global and local PDI methods, each actuator/sensor path can provide a damage source image. However, the information contained in one source image is very limited, and the image is easily interfered by noise. Therefore, an image fusion scheme is used to improve the signal-to-noise ratio, thereby enhancing the imaging quality of the final imaging result [25].

In detail, assuming there exist *N* pairs of actuator/sensor paths in stator bar, the compromised fusion [25] is used for global PDI result P1, which can be expressed as:

(11) $$P1=\frac{1}{N}\sum_{i=1}^{N}P_{1}(i)$$

where $P_{1}(i)$ is the DPP values of an individual sensing path that is calculated from Equation (2). The conjunctive fusion [25] is then used for the local PDI result P2, defined as:

(12) $$P2=\prod_{i=1}^{N}P_{2}(i)$$

where $P_{2}(i)$ is the DPP values of an individual sensing path that is calculated from Equation (8). The two above results P1 and P2 are normalized as $\bar{P1}$ and $\bar{P2}$, and the final imaging result *P* is given as:

(13) $$P=\sqrt{\bar{P1}*\bar{P2}}$$

The image fusion schemes averages all probability contributions, balancing the smallest and greatest probabilities and giving an intermediate measure of the existence of damage. It is beneficial for the suppression of noise interference.

## 3. Stator Insulation Damage Identification Experiment and Results

### 3.1. Experimental Setup

**Figure 3:**
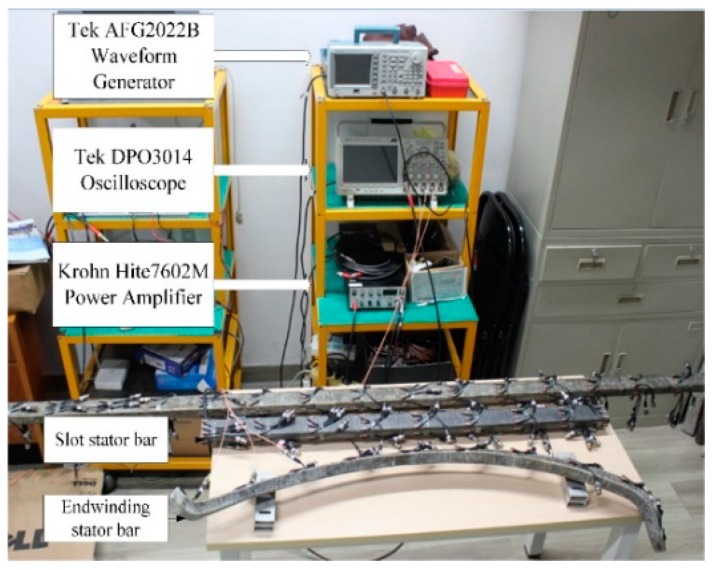
Setup of stator insulation damage detection test rig.

To validate the feasibility and performance of the proposed method, experimental work was carried out on real stator bar for insulation damage identification. The picture of test rig is shown in Figure 3. The specimens of stator bars are taken from a large generator rated 18 kV/300 MW. The cross sectional dimensions of stator bar is 60 mm × 30 mm and the thickness of ground wall insulation is 6 mm. The material parameters of stator insulation were tested as: Young’s modulus of 35 Gpa, density of 1720 kg/m^3^ and Poisson’s ratio of 0.2. Based on these parameters, the dispersive curves for Lamb wave propagation in stator insulation can be acquired by solving the Rayleigh-Lamb Equations [20,26]. In accordance with [20], the excitation signal in the experiment adopted a five-cycle Hanning-windowed sinusoidal toneburst with a central frequency of 15 kHz. The AFG3022B generates the preset toneburst excitation signal, followed by the 7602 M for power amplification. Such an amplified electrical signal can drive a PZT wafer to excite Lamb wave propagating in stator insulation. The PZT wafers (PIC161, PI) are adhered to the surface of stator insulation by using an epoxy adhesive. PZT wafers can serve as both the actuator and sensor of Lamb wave, respectively. The Lamb wave signal is then acquired by the DPO3014 with a sampling rate of 5 MS/s.

### 3.2. Performance Evaluation of ToF Extraction Methods

**Figure 4:**
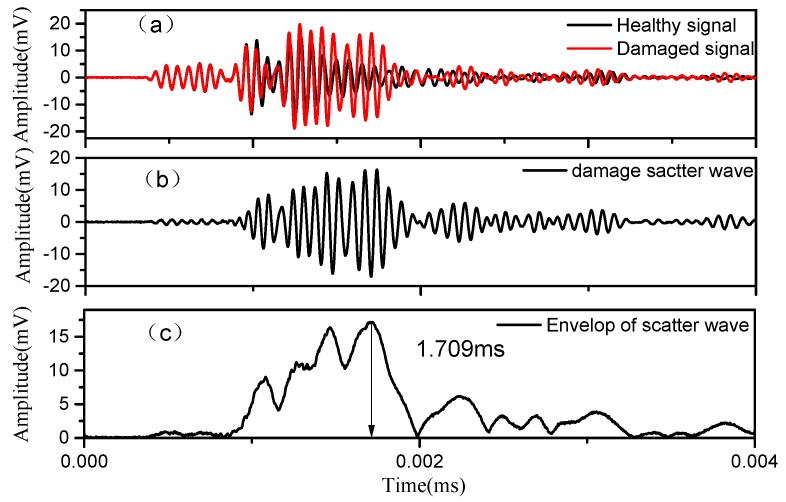
Time-domain Lamb wave signals in experiment: (**a**) healthy and damaged signal; (**b**) damage scatter wave; (**c**) envelop of scatter wave.

As discussed in Section 2.4, the ToF of damage scatter wave is a vital feature to indicate damage position, but it is prone to pollution by many factors. To evaluate the proposed WT-based ToF extraction method, the procedure of extracting ToF from Lamb wave signals by WT and Hilbert transform (HT) [20] method are first described. Figure 4a shows the time domain waveforms of Lamb wave signals in healthy and damaged states, respectively. The damaged signal appears different from its counterpart’s healthy signal, which indicates that the propagation of the Lamb wave on the stator bar has been impacted by the insulation damage. The difference between a healthy and damaged signal can be regarded as the damage-scatter wave [21,23], as plotted in Figure 4b. HT is carried out to acquire the damage-scatter wave envelope, and result is shown in Figure 4c. The ToF of scatter wave is then determined to be 1.509 ms, that is, the time delay between the envelope peak of scatter wave (1.709 ms) and the envelope peak of excitation wave (0.5 ms).

**Figure 5:**
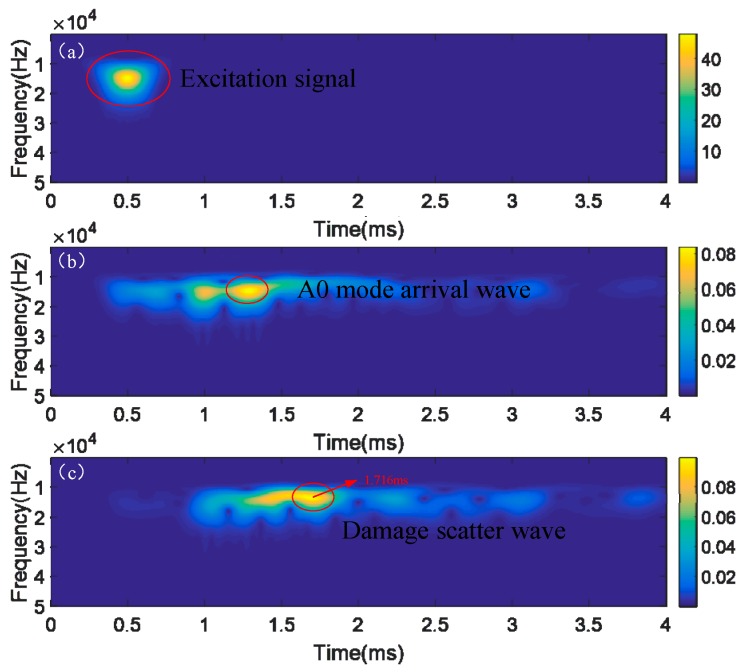
Wavelet energy spectrum for ToF extraction by WT method: (**a**) excitation signal; (**b**) incident wave; (**c**) scatter wave.

Figure 5 shows the wavelet energy spectrum of a series of Lamb wave signals after using WT. Db10 is adopted as wavelet function. The time-frequency distribution of the arrival wave and damage-scatter wave can be clearly seen in Figure 5b,c. The ToF of scatter wave is sensitive to the insulation damage position. In detail, the procedure to extract ToF of scatter wave is: at a center frequency of 15 kHz, the time at which the wavelet spectrum of scattered wave in Figure 5c reaches the maximum value is determined as the arrival time (1.716 ms). The beginning time of the excitation wave is derived as 0.500 ms from Figure 5a, similarly. The corresponding ToF (1.216 ms) is the time delay between the arrival time (1.716 ms) and the beginning time (0.5 ms).

**Figure 6:**
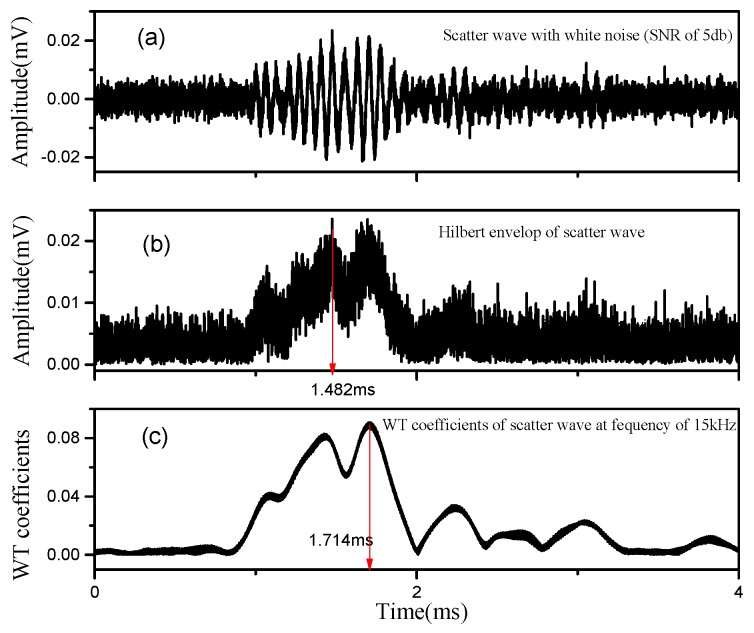
ToF extraction after adding white noise: (**a**) scatter wave with AGWN (SNR is 5 db); (**b**) ToF extracted by HT; (**c**) ToF extracted by WT.

**Figure 7:**
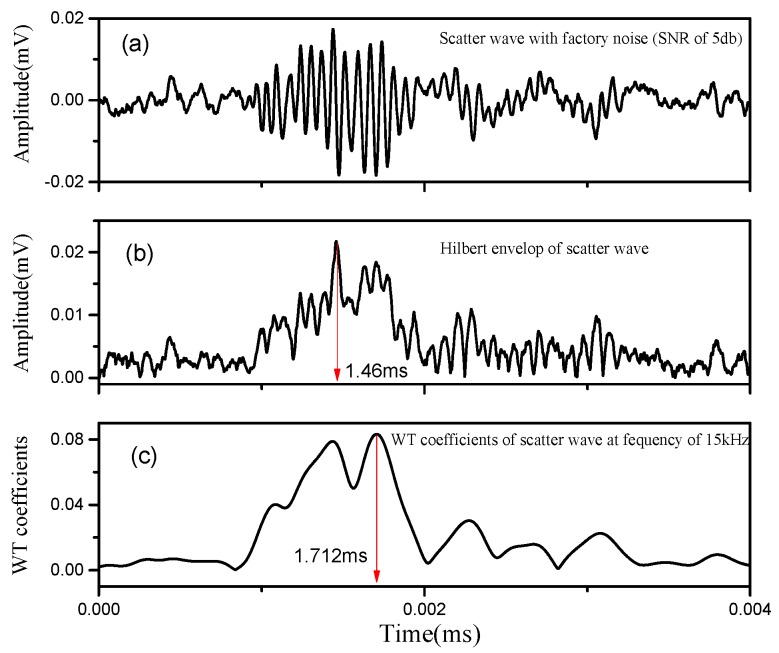
ToF extraction under FN environment: (**a**) scatter wave with FN (SNR: 5 db); (**b**) HT-based ToF extraction; (**c**) WT-based ToF extraction.

Then, noise robustness of the WT and HT method for ToF extraction is compared in the experiment. Figure 6 and Figure 7 show the ToF extraction results when the scatter wave is added with additive Gaussian white noise (AGWN) and factory noise (FN), respectively. The SNR in the two cases is both 5 db. In Figure 6, the arrival time of scatter wave determined by the HT method is 1.482 ms, while it is 1.714 ms by WT method. In Figure 7, the arrival time of scatter wave by HT and WT method are 1.460 ms and 1.712 ms, respectively. The HT method shows a nearly 0.2 ms deviation of ToF extraction under a noisy environment. In contrast, the WT method presents quite a high consistency even though the scattered wave signals are severely polluted.

The comparative ToF extraction results between the HT and WT methods under different noise conditions are shown in Table 1. The results indicate that the difference of HT and WT methods are small when the scatter waves have not been polluted by noise. However, the HT method is prone to being affected by noise with a decrease in SNR. The WT method outperforms than the HT method in anti-noise performance for ToF extraction, which can further improve the accuracy of damage localization, as exhibited in following section.

### 3.3. Identification of Puncture Damage in Stator Insulation

Previous research indicated that electric sparks might occur within air pockets in insulation by PD. These sparks contain electrons and ions that bombard mica-epoxy insulation. With enough time, the PD will erode a hole through the organic parts of the groundwall, leading to insulation puncture and causing catastrophic outcomes. Even a single insulation puncture will bring a very large circuiting current due to AC induction, and this high current will soon melt the copper conductors and adjacent insulation [5].

**Figure 8:**
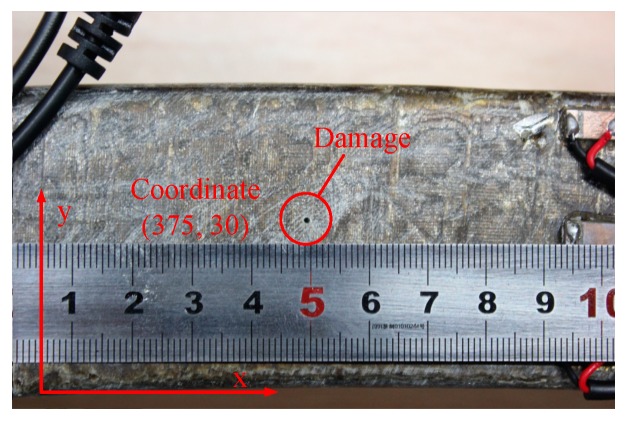
The puncture damage in ground-wall insulation.

To identify punctures in stator insulation by the proposed method, an insulation puncture damage with a depth of 6 mm and diameter of less than 1 mm was artificially made at coordinate (375, 30) of a 1200 mm length stator bar, as shown in Figure 8.

**Figure 9:**
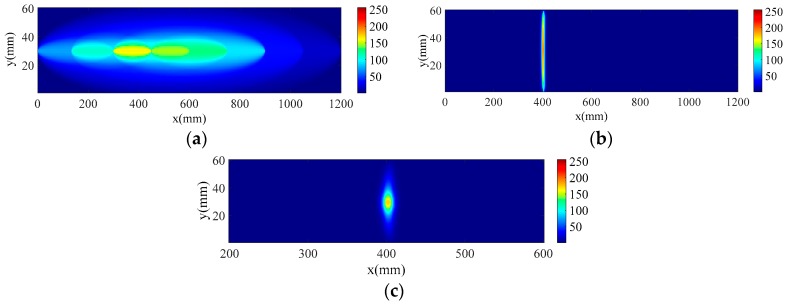
Identification results of puncture insulation damage using hierarchical PDI method. (**a**) Global PDI identification result; (**b**) Local PDI identification result; (**c**) Ultimate imaging result of stator insulation puncture damage.

Multi features of a Lamb wave related to the insulation damage were extracted from the PZT sensor network for probability imaging. Firstly, correlation coefficients between the healthy and damaged signal were extracted according to Equation (1); then, the DPP values were calculated by Equation (8), forming a color-scale image, and the global PDI result (Figure 9a) shows a preliminary area of damage distribution.

To further acquire the insulation damage location, local PDI is conducted based on the features of the damage scattered wave, including the maximum amplitude of scatter wave and ToF, which is extracted by WT method. In the PZT sensor network, the A0 mode scatter wave features are extracted for local PDI. The damage identification result is shown in Figure 9b. The global and local PDI result are fused according to Equation (13), and the ultimate imaging result is shown in Figure 9c. The puncture damage was identified at (402, 30) and the deviation between the identified damage location with actual damage location (375, 30) in x-direction is 27 mm. This shows that the position of puncture damage in stator insulation can be accurately located by the proposed hierarchical PDI method.

### 3.4. Identification of Crack Damage on Stator Insulation

**Figure 10:**
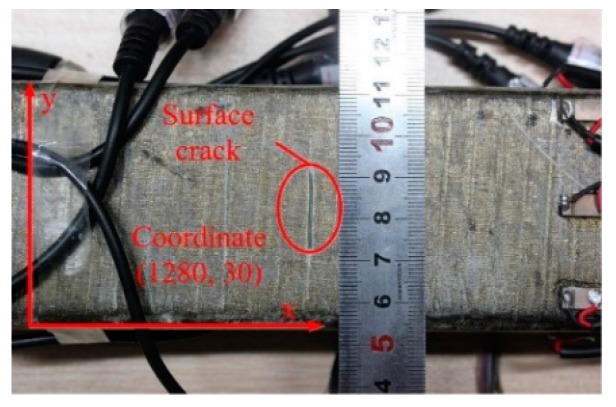
Crack damage on the groundwall insulation surface.

Electromagnetic forces and mechanical strain may cause mechanical fatigue, leading to cracks in the mica crystals and epoxy areas. On the other hand, thermal cycles can cause the tape layers to be separated radially (girth cracking) [4]. To evaluate the ability of the presented method in the identification of insulation crack damage, a 10 mm length crack (see Figure 10), was artificially made at coordinate (1280, 30) on a 2680 mm length stator bar.

**Figure 11:**
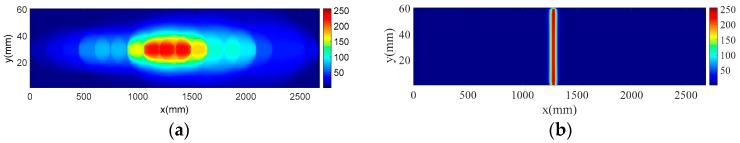

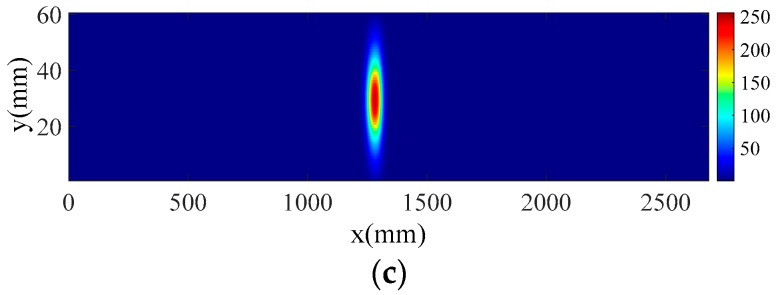
Identification results of crack insulation damage using hierarchical PDI method. (**a**) Global PDI result of crack insulation damage; (**b**) Local PDI result of crack insulation damage; (**c**) Ultimate imaging result of crack insulation damage.

Similar with the damage imaging procedure of puncture damage described above, the global PDI was first applied, and the corresponding imaging result is shown in Figure 11a. Then local PDI was conducted, and the imaging result is shown in Figure 11b. Image fusion was performed and the ultimate imaging result is shown in Figure 11c. From the image construction results, crack insulation damage was identified at coordinate (1293, 29). Compared to the actual damage position of (1280, 30), the location error in x-direction is 13 mm, presenting good damage location precision. The identification result of crack insulation damage in Figure 11 shows that not only the insulation damage location, but also the geometrical shape of the crack insulation damage can be identified in an intuitive and graphical manner.

## 4. Stator Insulation Inner Damage Identification Results

The inception of inner micro damage plays an important role in the final deterioration of stator insulation [10]. High electric stress may cause electrical breakdown in the insulation voids. Thermal aging accompanied with electrical force may cause molecular decomposition and oxidation of epoxy and mica in stator insulation [4]. As a consequence, delamination between layers of insulation is a common form of insulation damage [5], which would reduce the thermal conductivity of insulation and accelerate aging. Thus, to be of practical interest, it must be able to detect void and delamination damage in stator insulation. However, the inner insulation damage is difficult to simulate in experiment conditions.

**Figure 12:**
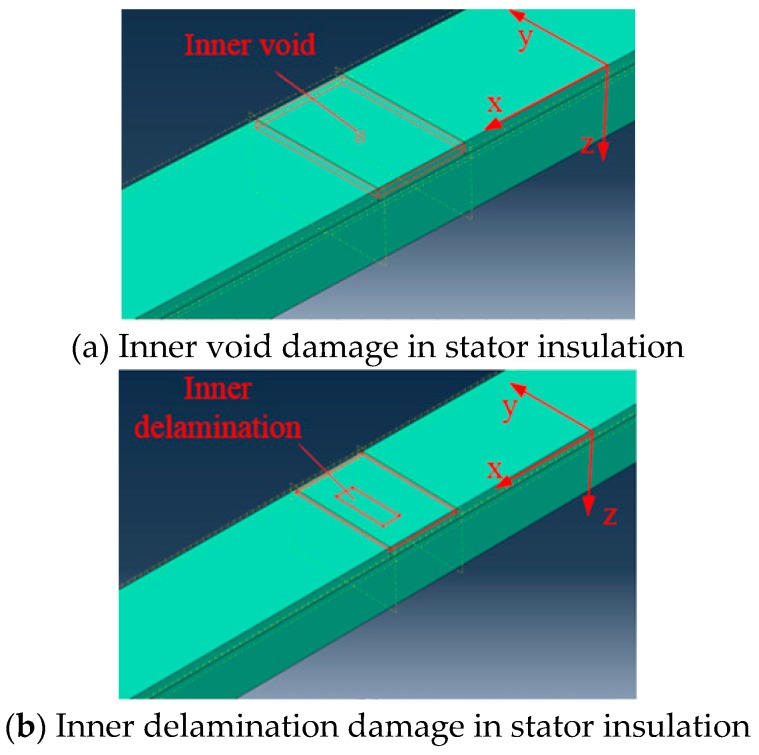
Structural damage models in stator insulation of FEM simulation.

The FEM software ABAQUS is a powerful tool to simulate the propagation property of Lamb wave in structure. The three-dimensional FEM model of a stator bar was established with geometry of 1200 mm in length (x direction), 60 mm width (y direction) and 30 mm height (z direction). An inner void (shown in Figure 12a) of 2 mm diameter and 1 mm height (z direction) inside stator insulation was simulated and configured at (825, 30). Similarly, an inner delamination (shown in Figure 12b) of 5 mm (x direction), 10 mm (y direction) and 1mm height (z direction) inside the stator bar insulation was made at coordinate (825, 30).

**Figure 13:**
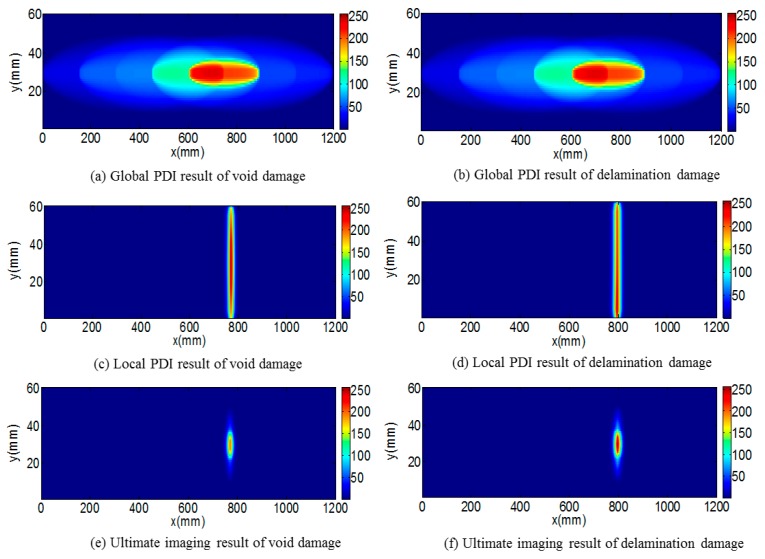
Procedure of hierarchical PDI for identification of void and delamination damage.

Figure 13 shows the imaging results of the inner void and delamination damage in FEM simulation by the proposed hierarchical PDI method. The existence and location of inner void and delamination damage in stator insulation can easily be identified. The identified inner void and delamination damage was located at (780, 29) and (800, 27), respectively. Location identification errors of void and delamination damage are 45 mm and 25 mm in x-direction, respectively. Considering the length of stator bar (1200 mm), these identification errors are quite acceptable.

To compare the proposed hierarchical PDI method using hybrid features of Lamb wave with the basic PDI method in [20], the location identification errors of the two methods are compared under a noisy environment (noise type: factory noise, SNR: 20 db). According to the comparative results, as listed in Table 2, the proposed PDI method shows a higher location identification precision of insulation damage under noise environment. In contrast, the basic PDI method in [20] is prone to be affected by noise interference; also, it is observed that the basic PDI method may fail to focalize the insulation damage with the decrease of SNR. Therefore, the proposed hierarchical PDI method presents good noise robustness with high localization precision.

## 5. Conclusions

In this paper, a hierarchical PDI method using hybrid features of Lamb wave is proposed to identify stator insulation damage of large generators with enhanced noise robustness and precision. The following conclusions can be drawn from the FEM simulation and experimental results:

- Using correlation coefficients as damage features, global PDI method can preliminarily determine the distribution area of groundwall insulation damage.
- Using the ToF and peak amplitude of the A0 mode damage-scattered Lamb wave as damage features, the local PDI method would further reflect the damage location in stator insulation.
- In contrast to the basic PDI method in [20], the proposed hierarchical PDI method shows a better anti-interference for feature extraction, which is more suitable for the field environment. Finally, the proposed PDI method is more accurate in graphically identifying both the location and geometrical shape of stator insulation damage. Therefore, it may provide a new detection method for CM method of large generator stator insulation.

## Figures and Tables

**Table 1:** ToF extraction results under different noise conditions.

Noise Condition (SNR)	ToF Extraction Method	
	WT Method	HT Method
Without noise	1.216 ms	1.209 ms
FN (10 db)	1.218 ms	1.052 ms
AGWN (10 db)	1.212 ms	1.070 ms
FN (5 db)	1.212 ms	0.940 ms
AGWN (5 db)	1.214 ms	0.982 ms

**Table 2:** Comparative results of insulation damage localization error by different PDI methods under noise environment (Factory noise, SNR is 20 db).

Insulation Damage Type	Insulation Location Error of Different PDI Methods	
	PDI Method in [20]	The Proposed PDI Method
Puncture	84 mm	38 mm
Crack	121 mm	16 mm
Inner void	failed to focalize	54 mm
Delamination	152 mm	35 mm
